# Correlative Light and Electron Cryo-Microscopy Workflow Combining Micropatterning, Ice Shield, and an In-Chamber Fluorescence Light Microscope

**DOI:** 10.21769/BioProtoc.4901

**Published:** 2023-12-20

**Authors:** Sabrina Berkamp, Deniz Daviran, Marit Smeets, Alexane Caignard, Riddhi A. Jani, Pia Sundermeyer, Caspar Jonker, Sven Gerlach, Bernd Hoffmann, Katherine Lau, Carsten Sachse

**Affiliations:** 1 Ernst-Ruska Centre for Microscopy and Spectroscopy with Electrons, ER-C-3/Structural Biology, Forschungszentrum Jülich, Jülich, Germany; 2 Institute of Biological Information Processing, IBI-6: Structural Cell Biology, Forschungszentrum Jülich, Jülich, Germany; 3 Delmic BV, Delft, the Netherlands; 4 Alvéole, Paris, France; 5 Institute of Biological Information Processing, IBI-2: Mechanobiology Forschungszentrum Jülich, Jülich, Germany; 6 Department of Biology, Heinrich Heine University, Düsseldorf, Germany

**Keywords:** CLEM, cryo-EM, Tomography, Cryo FIB–SEM, Fluorescence microscopy, Maskless micropatterning

## Abstract

In situ cryo-electron tomography (cryo-ET) is the most current, state-of-the-art technique to study cell machinery in its hydrated near-native state. The method provides ultrastructural details at sub-nanometer resolution for many components within the cellular context. Making use of recent advances in sample preparation techniques and combining this method with correlative light and electron microscopy (CLEM) approaches have enabled targeted molecular visualization. Nevertheless, the implementation has also added to the complexity of the workflow and introduced new obstacles in the way of streamlining and achieving high throughput, sample yield, and sample quality. Here, we report a detailed protocol by combining multiple newly available technologies to establish an integrated, high-throughput, optimized, and streamlined cryo-CLEM workflow for improved sample yield.

Key features

• PRIMO micropatterning allows precise cell positioning and maximum number of cell targets amenable to thinning with cryo focused-ion-beam–scanning electron microscopy.

• CERES ice shield ensures that the lamellae remain free of ice contamination during the batch milling process.

• METEOR in-chamber fluorescence microscope facilitates the targeted cryo focused-ion-beam (cryo FIB) milling of these targets.

• Combining the three technologies into one cryo-CLEM workflow maximizes sample yield, throughput, and efficiency.

Graphical overview

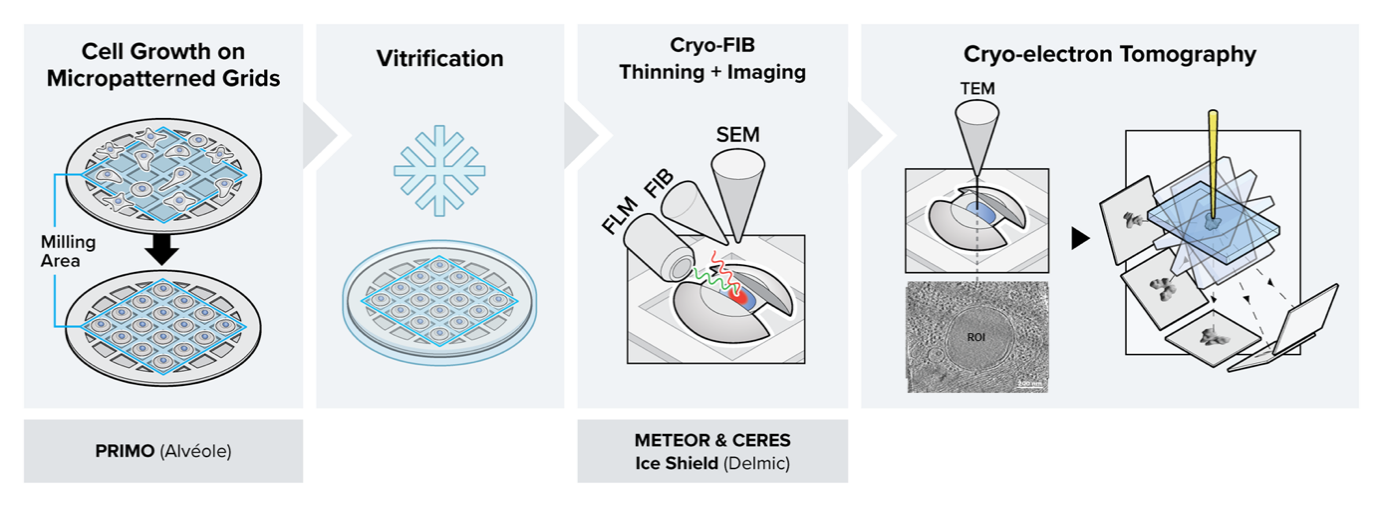

## Background

In the conventional in-situ cryo-electron tomography (cryo-ET) workflow, initially cultured cells expressing a fluorescent marker gene are required to adhere to an electron microscopy grid before they are cryo-fixed by plunge freezing (Iancu et al., 2007; Rigort et al., 2012; Mahamid et al., 2016). To navigate through the complex environment of the frozen-hydrated cell, correlative light and electron microscopy (CLEM) approaches were introduced into the workflow to localize the regions of interest (ROIs) (van Driel et al., 2009; Schorb and Briggs, 2014; Klein, Wachsmuth-Melm, et al., 2021). To this end, the grids are typically transferred to a cryo-fluorescence microscope to acquire spatial information on various features of interest using fluorescence (Arnold et al., 2016; Chakraborty et al., 2020). Following a second cryo-transfer step to a cryo focused-ion-beam–scanning electron microscopy (cryo FIB–SEM), the cells are thinned down to ~150 nm-thick lamellae using a focused ion beam, making them amenable to cryo-ET or cryo-EM imaging (Marko et al., 2007; Rigort et al., 2012; Villa et al., 2013; Lucas et al. 2021).

The workflow provides natively preserved insight into the structure-dependent functionality of cellular components at molecular or even close-to-atomic resolution (Xing et al., 2023). However, the complexity of the workflow poses challenges that frequently compromise the throughput and success rate (Hylton and Swulius, 2021). To name a few: i) conventional cell culture procedures lack proper control over cell shape and cell positioning on the grids. This limitation often results in cell clustering and cells positioned on grid bars rather than in the center of the grid squares, both of which make them unsuitable for FIB milling (Wagner et al., 2020). ii) The time-consuming nature of cryo FIB milling requires long residence time of the lamellae within the vacuum chamber. Consequently, the risk of in-chamber ice contamination significantly increases during batch milling sessions, making it impossible to mill multiple lamellae in an overnight session (Buckley et al., 2020; Tacke et al., 2021). iii) Combining CLEM approaches with cryo FIB milling allows for targeted preparation of lamellae at each intended region of interest (ROI). However, the cryo-transfer steps to and from the cryo-fluorescence microscope significantly increase the chance of ice contamination and devitrification of the sample. iv) The conventional way of lamella preparation and target determination is time consuming and complicated. Therefore, the error-prone procedure prior to lamella milling can easily lead to a mismatch in correlation and result in the loss of the ROI (Fukuda et al., 2014; Klein, Wimmer, et al., 2021).

In this protocol, we describe the details of a combined workflow with several newly available technological upgrades. i) Micropatterning the electron microscopy (EM) grids prior to cell culture using PRIMO: the customized patterns result in improved control over cell shape and spatial distribution over the entire grid (Engel et al., 2019; Swistak et al., 2021). By covering the EM grid in an anti-fouling layer and selectively removing it through UV light, the cell can only grow on selected areas of the grid. Provided the applied mask of the UV light is not too big, the cell will adapt to the mask shape, allowing one to reposition neuronal axons, study stress fibers in a specific area of

the cell periphery, or adjust the cell shape in some other way. Consequently, more millable sites will be available per grid, making an overnight milling session worthwhile (Toro-Nahuelpan et al., 2020; Sibert et al., 2021). ii) Cryo FIB milling in the presence of the CERES ice shield: the CERES ice shield eliminates the parasitic growth of an amorphous ice layer on top of the lamellae. Without it, lamella can be rendered unusable due to excessive ice contamination at the end of an overnight batch milling session (Tacke et al., 2021; Lau et al., 2022). iii) Continuous, in-chamber fluorescence detection using METEOR: by integrating the METEOR cryo-fluorescence microscope into the vacuum chamber of the cryo FIB–SEM, one major cryo-transfer step is eliminated from the workflow, which significantly reduces the chances of sample devitrification, contamination, and mishandling (Smeets et al., 2021). iv) The fluorescence integration in the FIB–SEM further allows for continuous monitoring of the ROI within the lamella during the milling process, thus eliminating the need for separate signal correlation to find back the ROI. This protocol has been proven to substantially increase the number of produced usable lamellae per milling session and hence surpass the conventional achievable success rate and throughput for high-resolution imaging in a cryo-transmission electron microscope (cryo-TEM). One can normally expect to produce 3–7 lamella in a fully manual milling session, and an estimated 25 lamella in automated session using micropatterned grids and the technological improvements described in this protocol, which are summarized in Table 1.

**Table 1.** Estimation of the effect of different workflow improvements on the output

	Estimated number of amenable milling sites per grid	Estimated number of final lamellae per grid
Manual milling, no micropatterning	8	~4
Manual milling + micropatterning	40	~8
Manual milling + micropatterning + CERES ice shield	40	~10
Automated milling + micropatterning + CERES ice shield + METEOR	40	~25

## Materials and reagents

**Biological materials**

1. Human immortalized RPE-1 cells (ATCC, hTERT RPE-1, CRL-4000), stably expressing human mCherry-p62

**Reagents**

1. 200 mesh gold Quantifoil grids R2/1 with SiO_2_ support layer (Quantifoil, catalog number: N1-S15nAu20-01, 100/pk)
2. PLL-g-PEG [SuSoS, catalog name: PLL(20)-g[3.5]- PEG(5)]
3. Poly L-Lysine (Sigma-Aldrich, catalog number: P8920)
4. mPEG-Succinimidyl Valerate MW 5,000 (mPEG-SVA) (Laysan Bio, Inc. catalog number MPEG-SVA-5000)
5. 14.5 mg/mL PLPP (1 mL PLPP solution) [Alvéole, catalog number: PLPP (for liquid) or PLPP_Gel (for gel)]
6. Fibronectin (human native fibronectin) (Gibco, catalog number: PHE0023)
7. Trypan blue (0.4% trypan blue stain) (Invitrogen, catalog number: T10282)
8. Trypsin (0.05% Trypsin-EDTA solution with phenol red) (Gibco, catalog number: 25300054)
9. Pen/strep (penicillin-streptomycin, 10.000 U/mL) (Gibco, catalog number: 15140122)
10. FCS (Fetal Bovine Serum) (Sigma-Aldrich, catalog number: F7524)
11. Phosphate buffered saline (PBS), pH 7.4 (Gibco, catalog number: 10010056)
12. DMEM (DMEM/F-12, GlutaMAX supplement) (Gibco, catalog number: 31331028)
13. Fluorobrite (FluoroBrite DMEM) (Gibco, catalog number: A1896701)

**Laboratory supplies**

1. Tweezers (Biological Tweezers, High Alloy DX Style 5) (Electron Microscopy Sciences, catalog number: 78325-5DX)
2. Parafilm (Parafilm M) (Sigma-Aldrich, catalog number: P7793)
3. Petri dish (900 mm Petri dish) (VWR, catalog number: 391-2016)
4. Primo stencil (PDMS stencil with 4 wells) (Alvéole, catalog number: PDMS_STENCILS_4)
5. 30 mm glass-bottom dishes (Cellview cell culture dishes, 35/10 mm, glass bottom) (Greiner Bio-One, catalog number: 627860)
6. T75 tissue culture flasks (T75 tissue culture flasks) (TPP, catalog number: Z707503-100EA)
7. Fluorescent marker (Staedtler, Textsurfer classic 364)
8. Black marker (black laboratory marker) (SecurLine, catalog number: 3083.2)
9. Cell counting slides (Countess Cell Counting Chamber Slides) (Invitrogen, catalog number: C10228)
10. Cell strainer (PluriStrainer mini 40 μm) (PluriSelect, catalog number: 43-10040-40)
11. 15 mL falcon tubes (15 mL conical centrifuge tubes) (Falcon, catalog number: 352196)
12. Eppendorf tubes (Safe-Lock 1.5 mL micro test tubes) (Eppendorf, catalog number: EP0030123328)
13. 1 μm FluoSpheres (FluoSpheres, carboxylate-modified microspheres) (Invitrogen, catalog number: F8816)
14. Ethane (Air Liquide, catalog number: P0500S10R0A001) or ethane/propane mix (37% ethane/63% propane, Nippon gas, catalog number: 611961)
15. Grid box for unclipped grids (Jena Biosciences, catalog number: X-CEM-201)
16. Grid box for clipped grids (Autogrid Compatible Cryo Grid Box) (MiTeGen, catalog number: M-CEM-7AGB)
17. Autogrid rings with cutout for FIB–SEM (Thermo Fisher Scientific, catalog number: 1205101)
18. C-ring (Thermo Fisher Scientific, catalog number: 103171)

## Equipment

1. Pelco easiGlow (Pelco easiGLOW Glow discharge Unit) (Ted Pella Inc. catalog number: 91000)
2. Pelco TEM grid holder block (Ted Pella inc., catalog number: 16820-25)
3. Fridge (Samsung, model: RL30J3005SA)
4. Inverted confocal or epifluorescence microscope (Nikon Eclipse Ti2 is used in this protocol)
5. PRIMO photopatterning device [Alvéole, Photopatterning Device consisting of a (DMD + laser 375 nm, 75 mW) Modulus, Driving Electronics, IHM software LEONARDO]
6. Mammalian cell incubator (Binder, catalog number: 9040-0112)
7. Laminar flow hood (Lamsystems, Uniflow, catalog number: 2E-B.003-15)
8. Water Bath (Julaba Pura 10, catalog number: 9 550 510)
9. Cell counter (Countess II automated cell counter) (Invitrogen, catalog number: AMQAX1000)
10. Widefield microscope (Zeiss Axio Vert.A1 FL)
11. Centrifuge (Eppendorf, model: 5702RH, catalog number: 5704000010)
12. Vitrobot Mark IV (Thermo Fisher Scientific)
13. Heating plate (CultureTemp 37 °C, Bel-art Products, catalog number: CHC7.1)
14. Clipping station (Thermo Fisher Scientific, catalog number: 1130697 or SubAngstrom product code CSA01)
15. Aquilos2 (Thermo Fisher Scientific)
16. CERES ice shield (Delmic B.V., catalog number: 2708-999-0010-1)
17. METEOR system (Delmic B.V., catalog number: 2707-999-0014-2)
18. High-end cryo TEM with direct electron detector for tomogram acquisition. Here, a 200 kV Talos Arctica G2 (Thermo Fisher Scientific) equipped with a Bioquantum GIF (Gatan) and K3 direct election detector (Gatan) was used

## Software and datasets

1. Inkscape or Adobe Illustrator (2020, version 24.1 used here)
2. Leonardo (version 4.15, https://www.alveolelab.com/our-products/leonardo-photopatterning-software/) (Access date, September 2023)
3. xT Microscope Control (version 20.1.1) (Access date, September 2023)
4. MAPS (version 3.19, https://www.thermofisher.com/order/catalog/product/de/en/MAPS2) (Access date, September 2023)
5. AutoTEM (version 2.4.1, https://www.thermofisher.com/order/catalog/product/AUTOTEM5?SID=srch-srp-AUTOTEM5) (Access date, September 2023)
6. ODEMIS (version 3.2.2, https://github.com/delmic/odemis) (Access date, September 2023)

## Procedure

- **Micropatterning of electron microscopy grids (Figure 1)**
  **Figure 1.**
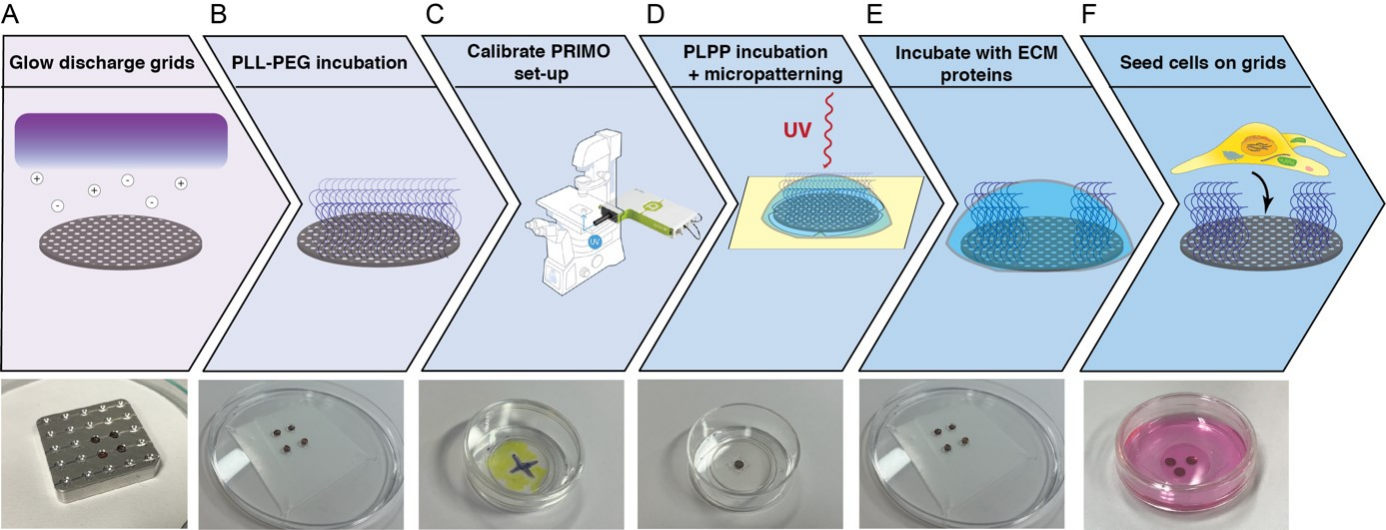
Overview of the micropatterning process. The electron microscopy grids are glow discharged to render them hydrophilic. (B) The grids are incubated in drops with PLL-g-PEG that acts as an anti-fouling layer. (C) The fluorescence microscope equipped with an Alvéole PRIMO device is focused and the DMD mirrors are calibrated. (D) The grids are incubated in PLPP, and the PLL-g-PEG layer is removed locally with a UV laser. (E) The grids are incubated with fibronectin or another extracellular matrix (ECM) protein. (F) Mammalian cells are seeded on the micropatterned grids.
  1. Grid passivation: application of anti-fouling layer on the grids. In this step of the process, the entire grid surface is covered in PLL-g-PEG, which prevents cell adhesion.
    - Take several 200 mesh gold Quantifoil R2/1 grids (SiO_2_ support layer).
    - Glow discharge both sides of the grid using a Pelco easiGlow plasma cleaner. Grids are placed in a holder or on a glass slide covered in parafilm for easier pick up and glow discharged at vacuum: 15 mA, 0.4 mBar, 60–90 s with 10 s hold. Notice the purple light indicating glow discharging is taking place. Flip over the grids with a tweezer to glow discharge the other side using the same settings. Glow discharging will allow the grid to be properly treated in the next steps, without the grid floating to the surface of droplets or sticking to the dishes used (Figure 1A).
    - Place the grids on a sheet of parafilm in a Petri dish with 30 μL drops of PLL-g-PEG, ~0.5 mg/mL in PBS, and incubate them overnight at 4 °C (Figure 1B).
    - Wash the grids by dipping them vertically in PBS several times.
    - Store the grids temporarily in a dish with PBS. The grids can be stored for several days at 4 °C.
  2. Micropatterning of grids using PRIMO photopatterning device and PLPP.
    - Turn on the fluorescence microscope and PC.
    - Draw the pattern for patterning in Inkscape or Adobe Illustrator. Save it as a .png file with 125 DPI.
    - Place an empty glass-bottom dish onto the microscope for calibration purposes. Mark the dish with a fluorescent marker and draw a small cross with a black marker.
    - Insert a 20× aperture (20× S Plan Fluor ELWD, 0.45 NA, Air is used in this protocol).
    - Focus the microscope on the black cross and set the perfect focus system or other autofocus system at this z-height (Figure 1C).
    - Move the dish slightly to an area with fluorescent marker, open the Leonardo software, and click on *Calibrate tube lens*.
    - Choose a calibration pattern from the options supplied in the Leonardo software. You can choose any pattern to calibrate the tube lens, but we recommend a pattern with features going down to 1 µm. Additionally, we recommend choosing a pattern that will resemble the pattern you will actually use for your experiment. If you will pattern lines, then choose a pattern with lines. If you will pattern dots or some exotic shapes, then choose to calibrate with dots. The Leonardo software contains basic calibration patterns to choose from.
    - Turn down the laser power and exposure time until you clearly see the smallest sub-structures in the pattern.
    - When the pattern is out of focus, turn the DMD wheel on the side of the PRIMO device.
    - On the next screen, the pixel ratio and the laser power are displayed. Confirm these are within expected values.
    - Stick a PRIMO stencil with 4 mm circular holes in a new 30 mm glass-bottom dish.
    - Pipette 2 μL of PLPP in a hole and ensure it spreads to cover the entire surface of the hole.
    - Take a grid and blot away excess PBS. **Critical:** Do not blot completely dry.
    - Place the grid in the PLPP droplet and replace the lid of the dish to prevent the grid from drying out and place the dish on the microscope. The orientation of the grid does not matter (Figure 1D).
    - Center the grid and focus on the Quantifoil film using the reflected light mode on the microscope.
    - Open your pattern file in the Leonardo software. The pattern contains a grid of white masks on a black background and determines the appropriate diameter of the shape for your cell type. The cell needs to have sufficient space to grow and spread, while ideally restricting the area to one cell per masked area (Figure 2). Move and rotate the pattern so that it is placed in the center of the grid squares (if desired) and at the center of the EM grid near the asymmetric mark. You may need to access the advanced features to enable pattern rotation. It is advisable to pattern only the squares in the middle of the grid for easier access with FIB milling and tomography.
      **Figure 2.**
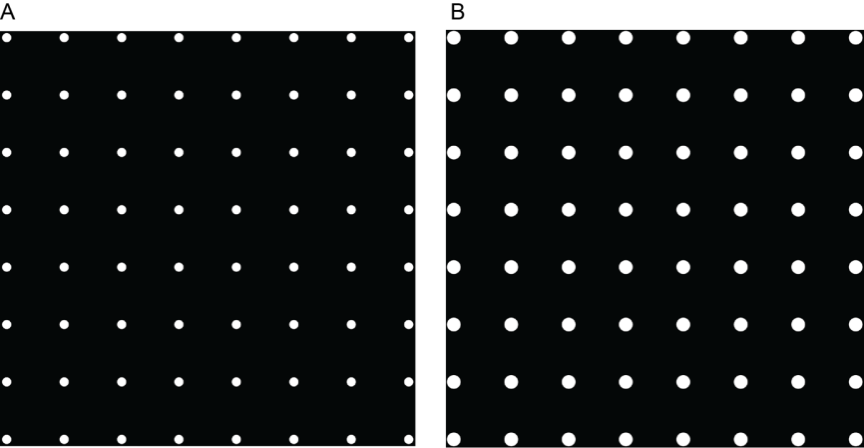
Examples of patterns of employed masks used on EM grids. A. An 8 × 8 grid of 20 μm circular masks used in the micropatterning procedure. B. An 8 × 8 grid of 30 μm circular masks used in the micropatterning procedure.
    - Set the dose to 900 mJ/mm^2 ^and enable stitching mode.
    - Switch the fluorescence microscope to the correct optical path, compatible with the PRIMO device.
    - Lock the pattern in place and press start to begin the micropatterning procedure. You should see small spots of light reflecting off the grid surface. This process should take 3–10 min, depending on the size of the pattern and the number of squares that you want to pattern.
    - Immediately remove the grid after the micropatterning procedure is done to avoid excess PLL-g-PEG layer degradation and wash the grid several times in fresh PBS.
    - Place the grids in a fresh 30 mm glass-bottom dish filled with PBS.
    - Wash the glass-bottom dish with the stencil used for micropatterning before repeating the protocol for the other grids.
    - **Pause point:** The micropatterned grids can be stored in PBS at 4 °C for up to four weeks. **Critical:** Be careful and avoid grids drying out.
**Side protocol—micropatterning of grids using PRIMO and PLPP gel:**
      Alternatively, the micropatterning of grids with PRIMO can be done using PLPP gel instead of PLPP (Engel et al., 2021). Indeed, the PLPP photoinitiator from Alvéole is also available in a gel form, which speeds up the photopatterning 30 times in comparison to the liquid form. Another advantage is that you can flip the sample upside down to be able to print on the EM grid bars if needed. The protocol using PLPP gel requires several drying steps of the grid, and the passivation is made with polylysine (PLL) and then mPEG-SVA:
      - As in the regular PLPP procedure described above, glow discharge and place a PDMS stencil on your grid.
      - Grid passivation: incubate your grid with a PLL solution (100 μg/mL for 30 min), rinse three times with 20 mM HEPES at pH = 8.5, and incubate your grid again with a solution of mPEG-SVA (10–20 μL for 1 h). Alternatively, an incubation with PLL-g-PEG can be done as described above.
      - Micropatterning with PRIMO: a drop of PLPP gel (1 μL) is diluted in deionized water (2 μL) and spread on the grid. After the complete drying of the solution, the grid is placed upside down on the microscope deck holder.
      - The steps using the Leonardo software are then similar to the PLPP protocol described above, with significantly lower UV doses (20–40 mJ/mm^2^).
  3. Incubation of grids with fibronectin.
    - Prepare a Petri dish with a small square of parafilm.
    - Place 30 μL drops of fibronectin (50 μg/mL) on the parafilm and place a grid on each drop. Incubate at room temperature for 20–30 min. The fibronectin solution was prepared by diluting the fibronectin stock solution in PBS.
    - Wash the grids twice in PBS for 5 min and once with DMEM before use.
    - **Critical:** Be careful and avoid grids drying out.
    - **Pause point:** The grids can be stored for up to seven days in PBS at 4 °C. **Critical:** Be careful and avoid grids drying out.
- **Seeding mammalian cells on micropatterned grids**
  The next steps are all performed under sterile conditions in a laminar flow hood.
  1. Preparing the cells for seeding.
    - Check the cells under a standard microscope for viability and cell health. Our cells were maintained in T75 flasks in 12 mL of DMEM media supplemented with Pen/strep and FCS.
    - Aspirate the media and briefly wash the cells with warm PBS.
    - Add 2 mL of 0.05% trypsin and incubate for several minutes at 37 °C with 5% CO_2_ until the cells detach from the dish.
    - Add 5 mL of DMEM, resuspend the cells, and pipette them in a 15 mL Falcon tube.
    - Centrifuge the cells at 500× *g* for 5 min at 37 °C.
    - Aspirate the supernatant and resuspend the cells carefully in a small amount of fresh, warm DMEM.
    - Pass the entire cell suspension through a 40 μm cell strainer into a new 15 mL Falcon tube to obtain single cells.
    - Mix 10 μL of cell suspension with 10 μL of Trypan Blue and pipette 10 μL of the mixture on a counting slide. Use the cell counter to determine the concentration of living cells in number of cells/mL.
    - Place the micropatterned grids in ~1 mL of DMEM supplemented with Pen/strep and FCS in a fresh 30 mm glass-bottom dish and carefully pipette 200,000 cells dropwise on top of the grids. Ensure the grids are on the bottom of the dish, Quantifoil film up, evenly spaced and a short distance away from the edges of the dish. Incubate the dish with the grids and the cells in the cell incubator at 37 °C with 5% CO_2_. Save the remainder of the cells in a warm place (Figure 1E).
      **Critical:** Optimize the cell density and incubation time for your cell line. Adding too many cells will lead to cells growing also on areas of the grid covered with an anti-fouling layer. Adding too few cells will lead to reduced throughput and loss of enough sites suitable for FIB milling.
  2. Assessment of the grids.
    - After 30–60 min, remove the grids from the incubator and look at them under a light microscope.
    - The cells should be attaching themselves to the areas of the grid that were micropatterned and to the areas of the dish surrounding the grids. When the micropatterned areas do not have a lot of cells, add additional cells dropwise and incubate again.
    - Incubate the cells on the grid 4–5 h before plunge freezing at 37 °C with 5% CO_2_.
- **Vitrification of mammalian cells**
  1. Turn the vitrobot on and warm the chamber to 37 °C with 85% humidity.
  2. Pre-warm some Fluorobrite medium on a small heating plate of the incubator and, when needed, make a suspension of 1 μm FluoSphere fiducials in Fluorobrite medium (1:30).
  3. Assemble and cool down the nitrogen container with liquid nitrogen. Once it is cold, fill the ethane container with ethane or ethane/propane mix. The ethane will turn cloudy and freeze on the sides of the cup when the cryogen is at the correct temperature.
  4. Place the grids, Fluorobrite medium for washing, and the fiducial suspension on a 37 °C hot plate or small incubator.
  5. Place the blotting paper on one blotting pad and a ring of parafilm on the other blotting pad for backside blotting.
  6. For each grid, carefully pick the grid up with the vitrobot tweezers by the outer ring, trying not to disturb the Quantifoil film. Attach the tweezers to the vitrobot with the top of the grid and the cells facing towards the pad with the parafilm. Briefly dip the grids in Fluorobrite to remove the autofluorescent DMEM medium.
  7. Add 3.6 μL of 1:30 FluorSpheres fiducial suspension right before plunge-freezing. Ensure the suspension is properly mixed by vortexing.
  8. Blot the grids for 8.5 s with 10 blot force and 1 drain time. As vitrobot machines are not entirely reproducible, the settings may need to be adjusted when needed to increase or reduce ice thickness. Yearly instrument alignment performed by Thermo Fisher is advisable to help with reproducibility. The blot force can be measured according to the protocol in Sader et al. (2020).
  9. **Pause point:** After plunge-freezing, store the grids in a grid box for unclipped grids in a liquid nitrogen dewar.
- **Thinning of the cells to ~150 nm using cryo FIB–SEM**
  1. Assessment of ice contamination rate in the FIB–SEM chamber.
*Notes: Chamber vacuum can affect the contamination rate. Installation of the Delmic CERES ice shield can reduce the contamination rate significantly. Assessment of the contamination rate under different conditions can help determine how long a user can mill the grid without the lamellae deteriorating to an unusable thickness (Figure 3).*
    **Figure 3.**
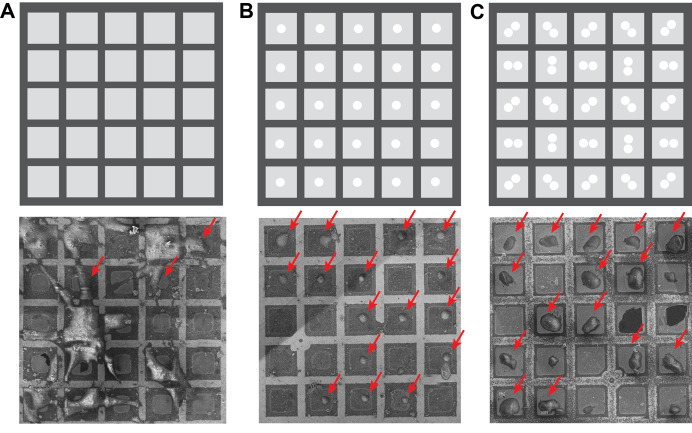
Micropatterning of EM grids can be used to greatly improve grid quality for cryo focused-ion-beam–scanning electron microscopy (FIB–SEM). A. Top: schematic of a grid pattern. Bottom: a typical EM grid that was incubated with human mesenchymal stem cells. B. Top: grid pattern with 20 μm diameter circles in the center of the grid square used for PRIMO photopatterning before seeding cells on the grid. Bottom: photopatterned EM grid with centrally adhered RPE-1 cells. C. Top: two touching 20 μm diameter circles were patterned in the center of all grid squares to align cell adherence to allow for milling from different angles. Bottom: an EM grid that was photopatterned with the PRIMO system. Possible milling sites are indicated with red arrows.
    - Load an empty, clipped grid into the FIB–SEM.
    - Navigate to an area in the center of the grid, focus, and determine eucentric height. Use a stage rotation of 110° and a stage tilt of 30°. For the full procedure, see details below.
    - Set the magnification to cover approximately 4 μm horizontal field width (HFW) and image with the electron beam. Focus on the film next to the holes. Use a scan rotation of 0°.
    - Pick two holes in the support film close to the top of the grid and measure the thickness of the film on the edge of the holes. Repeat this for two holes in the center of the grid and two holes close to the bottom of the grid.
    - Wait a fixed amount of time and repeat the procedure. Imaging the same area multiple times could shrink the built-up ice layer. To avoid this, after each timepoint, use a different set of holes to do the measurement.
    - Plot the data in a graph and calculate the slope to obtain the contamination rate.
  2. Selection and preparation of sites appropriate for milling.
    - Preparing the cryo FIB–SEM:
      i. *Wake up* the system in the xT microscope Control software.
      ii. In the *Beam Control* tab, select *Sputter Vacuum* and purge the lines five times. This should take approximately 20 min. Return to *High Vacuum* when done.
      iii. In the *Cryo TEM Preparation* tab, purge the GIS lines for 30 s.
      iv. Fill the dewar with liquid nitrogen and place the heat exchanger inside to cool down the system.
    - Clipping the grids:
      i. Follow Thermo Fisher’s procedure to clip the grids.
      **Critical:** Work carefully to avoid buildup of ice contamination on the grids.
      ii. Use special Auto-grid rings with a cutout to improve grid access. Mark the edges of the ring with a black or blue marker to help position the grids in the autoloader of the TEM microscope.
      iii. Save the grids in a grid box for AutoGrids under liquid nitrogen.
    - Loading the grids into the FIB–SEM:
      i. Cool down the loading station and insert a pre-tilted shuttle.
      ii. Once it is cooled down, place the grid boxes into the station and load two grids into the shuttle. Ensure that the Autogrid cutout is positioned perfectly at the top. Work carefully to avoid buildup of ice contamination on the grids.
      iii. Quickly load the shuttle into the FIB–SEM chamber and check proper position using the in-chamber camera before disconnecting the transfer rod.
    - Atlas acquisition:
      i. Move the shuttle to the mapping position in the chamber using the xT Microscope Control software. It may help to set the scan rotation for the ion and electron beams to 180° for easier visualization.
      ii. Using the SEM beam and the Everhart-Thornley detector (ETD), focus the beam on the grid and correct for astigmatism. Use a 2 kV beam at 25 pA for samples with thin ice and a 5 kV beam for samples with thicker ice.
      iii. Open MAPS software and take a snapshot of the whole grid.
      iv. Place a tile set on the center of the grid, 5 × 7 tiles with a tile height of 600 μm HFW and a total HFW of 2.76 mm. Set the resolution to 3,072 × 2,048, the pixel size to 195 nm, and the dwell time to 1 us. Start the acquisition.
    - Eucentric height and milling angle determination:
      i. Mark the potential milling sites in the MAPS software by clicking *Add Lamella Site Here*. Ideally, these are single cells that are positioned in the middle of the grid squares, without any ice contamination on top. Only squares close to the center of the grid should ideally be selected, as the sites close to the Autogrid ring will yield small lamellae due to a high milling angle.
      ii. Navigate to each site using the *Drive To Mapping Position* button, increase the magnification of the SEM beam, and focus the beam on the carbon film. Use 2–5 kV, 13 pA, and a 300 ns dwell time. While in live mode, *link z*.
      iii. Determine the eucentric height of each milling site using a manual approach; the prompts are available in MAPS or the automated procedure is available in AutoTEM. Turn on the ion beam and use the ETD detector to image each site. Focus the beam. Use 30 kV, 30 pA, and 100 ns dwell time. Tilt the stage to 20° and image the site. If the edge of the Autogrid ring is in view, increase the tilt angle. When it is not in view, decrease the angle. From the point where the edge of the Autogrid ring is in view, tilt up 1–2° to get sufficient clearance for the ion beam not to be deflected. Mark this milling angle in MAPS software by clicking *Store Angle*. The icon next to the name of the lamella site will change from orange to green.
      iv. Mark each site at the eucentric height and the milling angle in the FIB–SEM GUI for easier navigation later.
  3. Assessment of milling sites by the METEOR system.
    - Make sure the z-height of the stage is linked in the microscope GUI.
    - Open the ODEMIS software on the METEOR microscope PC and make a new project folder. Press *FM imaging* to move the stage to the METEOR position and insert the objective lens (LMPLFLN 50×/NA = 0.5/WD = 10.6 mm).
    - Add imaging streams for each fluorophore, select the power for each channel, and set the exposure time to ~300 μs. Usually, 200–400 mW is sufficient, depending on the fluorophore, the abundance of the ROI, and the camera on the system. Focus the image using the autofluorescence signal in the green channel by slowly approaching the sample with the objective lens until the signal inside the target cell is sharp or until you can see the holes in the Quantifoil support film due to its autofluorescent properties.
      **Critical:** Do not insert the objective lens too far, as it can crash into the shuttle and damage the lens.
    - Acquire an image of each lamella site to confirm the presence of your ROI in the cells. A good start for camera settings is: bin 1, resolution 1,190 × 928 px, a gain of 16-bit, and a readout rate of 310 MHz. Adjust the exposure time and the power in the rest of the channels to a value that will yield a clear signal but not an oversaturated histogram.
    - Save these imaging positions in the FIB–SEM xT Microscope Control software for easier navigation later.
    - Switch back to the milling site in the chamber by pressing *SEM imaging*. Wait for the objective lens to retract and the shuttle to move back to the milling position in the chamber.
    - Navigate to the next milling position using MAPS or the saved positions in the xT Microscope Control software and repeat Steps D3d–D3f.
  4. Setup of batch milling protocol.
    - Open AutoTEM. It should read in all the positions from the MAPS software.
    - Apply a template with the following parameters (Table 2) to all sites.
      **Table 2.** Milling parameters used in AutoTEM. Description of all parameters that were imported in AutoTEM and used in this protocol. Sample-dependent optimization may be required.

Preparation	Ion HFW Oversize: 80 μm Eucentric Tilt: disabled Artificial Features: disabled Milling Angle: disabled Image Acquisition: disabled Lamella Placement: enabled Ion HFW Oversize: 150% Minimal Ion HFW: 10 nm
Milling	Lamella Size: 17 μm × 3 μm Correction Factor: 0.60 Delay: disabled Reference Definition: enabled Electron Reference Definition: disabled Stress Relief Cuts: test if cuts help prevent lamella bending in your sample Reference Redefinition 1: disabled if no Stress Relief Cuts were made, otherwise enable Rough Milling: Pattern Offset: 1 μm Front Pattern Height: 7.2 μm Rear Pattern Height: 6.3 μm Depth Correction: 30% Front Width Overlap: 1.5 μm × 1.5 μm Rear Width Overlap: 1.5 μm × 1.5 μm Milling Current: 0.5 nA Pattern Type: Rectangle DCM Rescan Interval: 120 s Show Graphics: enabled Rough Milling - Electron Image: 1,536 × 1,024, 3 μs Enable ACB Enable Auto Focus HFW: 70 μm Reference Redefinition 2: enabled Medium Milling: Pattern Offset: 600 nm Front Width Overlap: 650 nm × 650 nm Rear Width Overlap: 650 nm × 650 nm Overtilt: 0° Depth Correction: 160% Milling Current: 0.3 nA DCM Rescan Interval: 90 s Pattern Overlap: 400% Pattern Type: CleaningCrossSection Medium Milling - Electron Image: 1,536 × 1,024, 3 μs Enable ACB Disable Auto Focus HFW: 70 μm Fine Milling: Pattern Offset: 300 nm Front Width Overlap: 350 nm × 350 nm Rear Width Overlap: 350 nm × 350 nm Overtilt 0° Depth Correction: 160% Milling Current: 0.1 nA DCM Rescan Interval: 60 s Pattern Overlap: 200% Pattern Type: CleaningCrossSection Fine Milling - Electron Image: disabled Finer Milling: Pattern Offset: 200 nm Front Width Overlap: 50 nm × 50 nm Rear Width Overlap: 50 nm × 50 nm Overtilt 0° Depth Correction: 140% Milling Current: 50 pA DCM Rescan Interval: 30s Pattern Overlap: 200% Pattern Type: CleaningCrossSection Finer Milling - Electron Image: disabled
Thinning	Disabled
    - Navigate to each milling position by clicking the saved position in the xT Microscope Control software, focus both beams, and click *Update* in the AutoTEM GUI. The software will take an image and store the stage position and focus parameters for later use.
    - Enable the sites in the Milling List one by one. For each site, click *Run*, enable only the *Preparation* section of the protocol, and click *Ok*. AutoTEM will take an image and place a milling box in the center of the image according to the specifications in the template. Adjust the size and shape of the milling box and place them in the middle of the cell, with the center positioned where your ROI is. Note that some part of the cell should remain on either side to maintain the lamella. Press *Ok* to confirm the site and save it in the program. Navigate to the next site in the xT Microscope Control software and repeat the steps D4c–D4d.
    - Once all sites are marked and appear green in AutoTEM, apply a GIS layer on the grid for 8 s in a FIB–SEM without lift-out system or 90 s for a system with a lift-out system. The layer deposition is initiated by navigating to the *Cryo TEM preparation* tab in the xT Microscope Control software, selecting the grids you want the GIS to be deposited on, setting the Flow Duration, and clicking *Start*.
    - Fill the dewar of the FIB–SEM to ensure sufficient liquid nitrogen is present for the automated milling process to finish.
    - Click *Run* in AutoTEM. In the pop-up window, only enable the *Rough Milling* tab and make sure all sites are clicked. Disable *Fine Milling* when you plan on doing the lamella polishing manually. Set the program to mill step-wise and start the program.
    - Once you start the automated rough milling procedure, the CERES ice shield should automatically insert and stay inserted during all milling steps.
  5. Assessment of lamella quality with METEOR system.
    - Move the stage to the imaging position and insert the objective lens (LMPLFLN 50×/NA = 0.5/WD = 10.6 mm).
    - Reuse the same imaging streams for each fluorophore as desired and set the exposure time to ~300 μs. Focus the image using the autofluorescence signal in the green channel by slowly inserting the objective lens.
    - Take an image of each lamella site to confirm presence of your ROI in the lamellae using an exposure time that will yield a clear signal, but not resulting in an oversaturated histogram.
    - By using the saved imaging positions (step D4c) in the xT Microscope Control software, switch directly to the next imaging site. Make sure to retract the objective lens 5–10 mm before executing the stage movement.
    - Once all lamellae are imaged, move the shuttle back by pressing *SEM imaging* in the ODEMIS software.
  6. Polishing the lamella.
    - In AutoTEM, navigate to each site individually and manually inspect to confirm the presence of a good lamella. Focus the beam.
    - Polish the lamella step-wise by using a 30 kV ion beam at 30 pA using two rectangular boxes placed ~300 nm apart in the xT Microscope Control software. Regularly monitor the lamella and look for cracks, holes, bending, and a disappearing GIS layer on the leading edge of the lamella. It can be helpful to enable iSPI in xT Microscope Control and set it to automatically take an SEM image every 2–8 s. More SEM images will damage the lamella, so be conservative.
    - Move the boxes in closer to ~200 nm and repeat the process.
    - When the lamella remains intact and stable, lower the beam current to 10 pA, bring the boxes closer to ~150 nm or less, and repeat the thinning process.
      **Critical**: If the leading GIS layer starts degrading, it can be helpful to continue thinning using an overtilt. Disable the lower milling box and tilt the stage an additional +0.5°–1°. Mill only from the top at a 10 pA current. When the back of the lamella starts degrading or the GIS disappears, stop the milling process.
    - Appropriate lamella thickness can be estimated by taking two images with the SEM. Take an image at 5 kV and one at 2 kV. At around 250–300 nm lamella thickness, this lamella will appear dark gray in the 5 kV image but white or light gray in the 2 kV image. When the lamella thickness approaches 150–200 nm, the lamella will appear black in the 5 kV image and dark gray in the 2 kV image.
  7. Final image acquisition with METEOR system.
    - Move the stage to the imaging position and insert the objective lens (LMPLFLN 50×/NA = 0.5/WD = 10.6 mm).
    - Reuse the same imaging streams for each fluorophore as desired and set the exposure time to ~300 μs. Focus the image using the autofluorescence signal in the green channel by slowly inserting the objective lens.
    - Take an image of each lamella site to confirm presence of your ROI in the final lamella using an exposure time that will yield a clear signal, but not resulting in an oversaturated histogram. This may require an exposure time of up to 30 s in case of a small ROI or a small amount of fluorophore present.
    - By using the saved imaging positions in the xT Microscope Control software, switch directly to the next imaging site. Make sure to manually retract the objective lens 5–10 mm before executing the stage movement.
    - Once all lamellae are imaged, export all images as “print-ready tiffs” after adjusting the histogram outliers to get the final image.
  8. Sputter coating the lamella.
    - A thin platinum layer is added on top of all lamellae to increase lamella stability and reduce sample charging in the TEM microscope. Click *Prepare For Sputtering* in the xT Microscope Control software in the Cryo TEM Preparation tab.
    - Once the chamber pressure reaches 0.1 mbar, the *Sputter* button will appear and a sputter chamber will cover the shuttle, which has moved to a different chamber position.
    - Sputter for 5–7 s at 7 mA. Confirm sputtering is taking place by looking out for a glowing white light on the in-chamber camera.
    - Immediately after the sputtering is done, press *Recover From Sputtering* to return to high vacuum.
  9. Unload the grids.
    - Cool down the loading station.
    - Quickly remove the shuttle from the FIB–SEM chamber and place it in the loading station using the transfer rod. Take care not to slam the shuttle on the bottom of the loading station.
    - Flip the shuttle 90° and move the grids very carefully to a grid box for clipped grids. Work carefully to avoid buildup of ice contamination on the grids.
    - Store the grid box under clean liquid nitrogen until ready for TEM imaging.
    - Warm up the FIB–SEM chamber to room temperature by removing the heat exchanger from the liquid nitrogen dewar and “sleep” the system.

## Data analysis

**FM/SEM image correlation**

Correlation between FM and SEM images was performed using MATLAB R2021b (Natick, Massachusetts: The MathWorks Inc.) Briefly, sets of control pairs were chosen on both images based on recognizable features, and a similarity transformation matrix was determined using this point-to-point correspondence between FM and SEM images. The best transformation was determined using least squares minimization of a suitable cost function.

**Quantification of imageable areas within lamellae**

Quantification of imageable areas was performed using ImageJ software [US National Institutes of Health, Bethesda, Maryland, USA (Schindelin et al., 2012)]. Briefly, the TEM overview images of the lamellae were initially binned by a factor of 3 to allow for better visual differentiation of clean and contaminated regions within the lamellae. An initial global thresholding was performed to outline the major regions contaminated with thickest ice crystals (lowest gray values). The minor ice-contaminated regions were then manually selected using the free selection tool and the area corresponding to each region was calculated.

**Reconstruction of the tomograms**

Tilt series were motion corrected and CTF corrected using WARP (Tegunov and Cramer, 2019). Next, the tomogram was reconstructed using AreTomo (Zheng et al., 2022) and deconvolved and denoised using IsoNet (Liu et al., 2021). Visualization of the data was done using IMOD (Mastronarde and Hel, 2017).

## Validation of protocol

**Micropatterning of electron microscopy grids improves cell positioning and cell shape**

Figure 3 displays the effect of photopatterning with the PRIMO system and the associated benefit on grid quality. Figure 3A displays a traditional, non-micropatterned grid. Note that the cells clump together leading to poor vitrification. Many cells are also positioned on top of the grid bars, making them inaccessible for thinning with the cryo FIB–SEM. Figure 3B shows an EM grid that was photopatterned. Cells are perfectly positioned in the center of the grid squares, leading to a significant increase in possible FIB milling sites, as indicated with the red arrows. Figure 3C reveals how cells can be deliberately aligned to cover all possible milling orientations. A modified pattern can be used to help reposition ROIs or organelles to an area of choice.

**Micropatterning of electron microscopy grids can aid in ROI positioning to the center of the grid squares**

In many cases, the cellular feature of interest will be scarce and may be located far away from the cell nucleus, which complicates reliable positioning of the ROI at the periphery of the cell.

**Figure 4.**
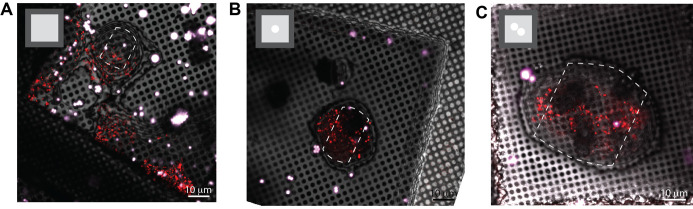
Micropatterning enables organelle positioning for ROIs located at the periphery of the cell. A. A typical EM grid that was incubated with RPE-1 cells. B. EM grid that was photopatterned with 20 μm diameter circles in the center of all grid squares before seeding RPE-1 cells on the grid. C. An EM grid that was photopatterned with two touching 20 μm diameter circular patterns in the center of the grid square. Possible milling sites are indicated with a white dotted line and the photopattern used is indicated in the upper left side of each image.

Figure 4 illustrates how micropatterning can be used to help re-direct the ROI to the center of the grid squares, improving the ability to target these cellular features. In Figure 4A, a grid that did not undergo micropatterning is shown. Note that there are several cells in this grid square, with the largest portion of the cell body positioned on or near the grid bars. Most of the ROIs are not accessible due to their proximity to the edges of the grid squares or due to their proximity to the thinner regions of the cell near the plasma membrane. In Figure 4B, a simple, circular pattern was used to help re-position the cells, but not the organelles of interest. Here, the cell is positioned away from the grid square edges. The ROI that was labeled with mCherry mainly localizes to the cell periphery, making it hard to capture in the lamella, as indicated with a white, dotted line. In Figure 4C, a modified pattern was used to increase the number of ROIs that could be captured in a lamella. Most of the cells had these features in the cell periphery. Therefore, the interface between the two cells was directed towards the center of the grid square by the photopattern. The lamella could now be positioned at the cell periphery, increasing the likelihood that the ROI (in red dots) could be captured in a single lamella. Other patterns can be designed to specifically position other organelles or cellular features.

**CERES ice shield reduces the ice contamination growth on the sample**

**Figure 5.**
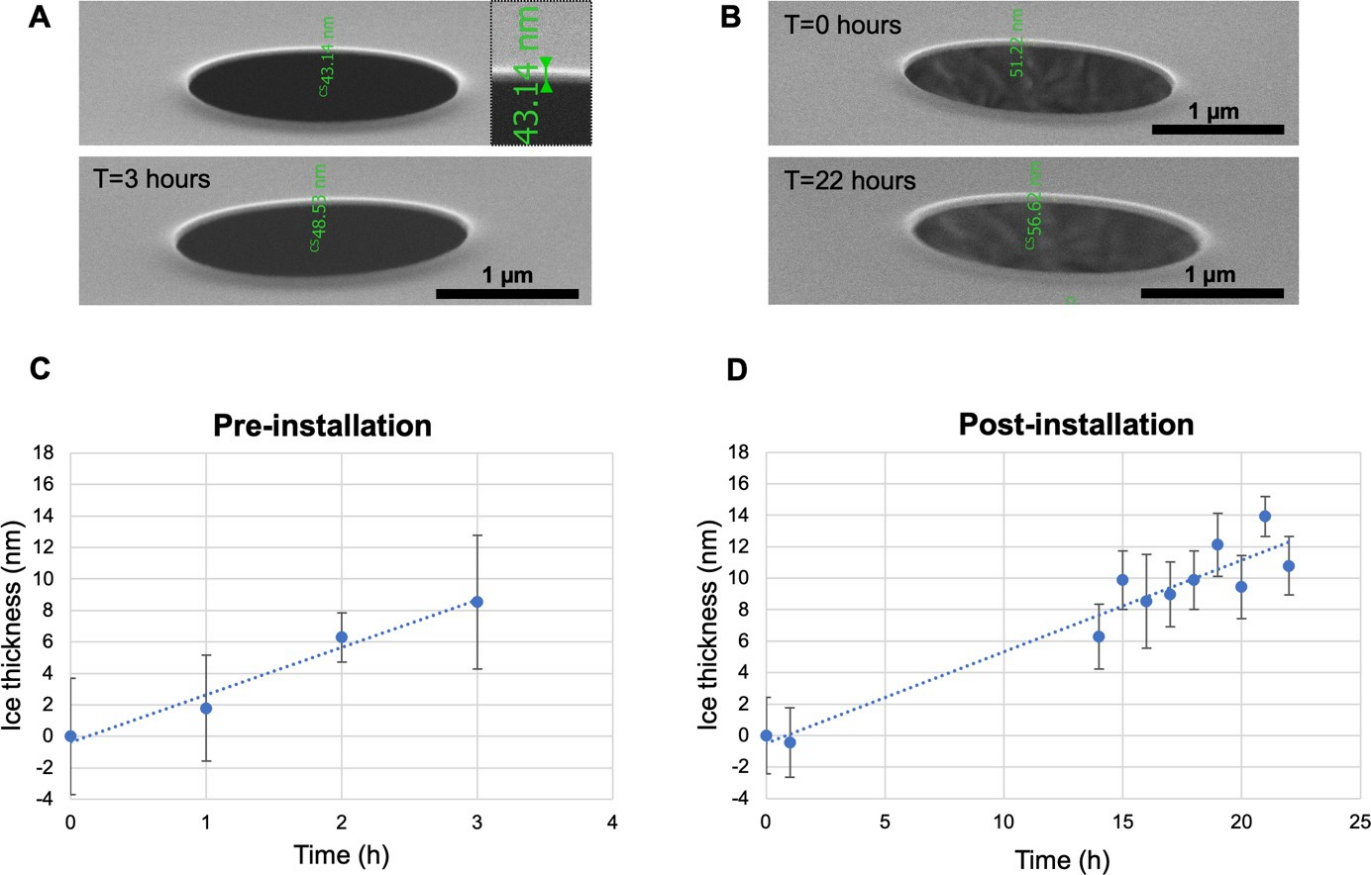
Determination of in-chamber ice growth rates before and after the installation of the CERES ice shield. A/B. Representative SEM images of Quantifoil holes on support used to measure the thickness of the ice layer: top row images depict the first time point (T = 0 h) and bottom row images depict the last time point (T = 3 h pre-installation, T = 22 h post-installation). Top right magnified inset shows the measurement of layer thickness on the image. To determine ice thickness, the layer thickness difference to T = 0 h was used. C/D. Corresponding diagrams of ice thickness at each measured time point before and after installation of the CERES ice shield. Measurements were done in triplicate on different grid regions to generate an average. The average ice growth rate was estimated by linear fitting at 3.0 and 0.6 nm/h before and after installation.

Figure 5A and 5B display representative SEM images illustrating the ice thickness estimation procedure described in Section D1. The results of these measurements were used to quantify the rate of in-chamber ice growth. The graphs in Figure 5C and 5D display the average thickness of the ice layer at each measured time point determined in triplicates before and after installation of the CERES ice shield, respectively. Before installation, the measured ice layer thickness after 3 h was approximately 8.5 ± 4.2 nm. By contrast, extending the residence time of the grids inside the chamber after installation and measuring the ice layer thickness after 22 h yielded an approximate ice layer thickness of 10.8 ± 2.0 nm. Based on these results, the average ice growth rate was estimated as 3.0 nm/h and 0.6 nm/h before and after installation of the CERES ice shield, respectively. The differences correspond to a >5-fold reduction in the ice contamination in the presence of the CERES ice shield. Please note that this Aquilos2 cryo FIB–SEM instrument already underwent chamber vacuum improvements leading to a lower contamination rate prior to the installation of the CERES ice shield. Older, similar devices showed an ice growth rate around 50 nm/h, which will drop below 2.0 nm/h after installation of the CERES ice shield (Tacke et al., 2021)

**The improved workflow using CERES ice shield and the METEOR system reduces ice contamination on lamellae and thereby greatly enhances sample throughput**

**Figure 6.**
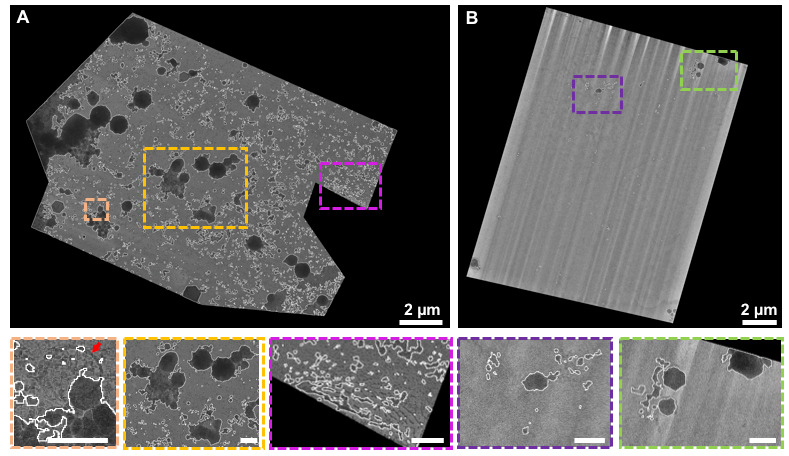
Extent of frost contamination on lamellae with and without in-chamber fluorescence detection. A. Overview TEM image of a batch-milled lamella where the confirmation of the ROI was performed with a separate cryo-confocal microscope. Due to the additional transfer step to the cryo-confocal microscope, ice crystals with different sizes and shapes (marked in white) have covered 25.4% of the shown lamella area. In the close-ups, the red arrow points toward an obscured potential ROI that otherwise could have been a candidate for tomogram acquisition. B. Overview TEM image of a batch-milled lamella where the presence of the ROI was confirmed within the vacuum chamber using METEOR. Eliminating the extra final transfer step to a separate fluorescence microscope led to clean lamella with 99.2% of imageable area (inset scale bars: 500 nm).

Figure 6 depicts the impact of ROI confirmation with METEOR compared to using a stand-alone cryo-fluorescence microscope. Without METEOR, the batch-milled lamellae were transferred to a cryo-confocal microscope to confirm the presence of ROIs. We used a Zeiss LSM 800 upright confocal microscope equipped with a Linkam CMS196 cryo-stage for this work, but any cryo-capable fluorescence microscope can be used to image the lamella. The contamination build-up rate will vary across different microscope and cryo-stage configurations, as well as handling speed and room humidity. As shown in the overview TEM image as well as the higher magnification close-ups in Figure 6A, this extra transfer step leads to significant crystalline ice contamination in the form of many large (up to 1 μm) opaque ice crystals across the lamella, obscuring the potential candidate regions for tomogram acquisition. The additional transfer puts the sample in further danger of devitrification. However, assessing lamella quality using METEOR in the FIB chamber, as described in Section 5, eliminates this extra transfer step and thereby results in clean, frost-free lamellae as displayed in Figure 6B. To quantify the impact of METEOR on reducing crystalline ice contamination, TEM overview images of six lamellae without use of METEOR and nine lamellae with use of METEOR were screened for detecting the crystalline ice particles. Without using the integrated METEOR in the workflow, 30.2% of obscured area was determined in 668 μm^2^ of total screened area. With METEOR, 2.8% of obscured area was detected in 2200 μm^2^ of screened area. The presence of the CERES ice shield and the METEOR system enabled more intact lamellae to be milled (+329% increase in total surface area) and led to more usable area per session (+458% increase).

To validate the effectiveness of the upgraded workflow, the described procedure was tested on stable, immortalized human RPE-1 cells expressing mCherry-p62. p62/SQSTM1 (hereafter, p62) is a classic autophagy cargo receptor that binds poly-ubiquitinated cargo at its C-terminus and polymerizes into filaments at its N-terminus (Ciuffa et al. 2015; Jakobi et al. 2020). This polymerization results in phase separation showing droplets within the cell, thereby facilitating autophagic cargo concentration and segregation (Turco et al., 2021). The droplets are subsequently confined in a double membrane forming an autophagosome. Finally, the autophagosomes are transported to the lysosomes.

**Figure 7.**
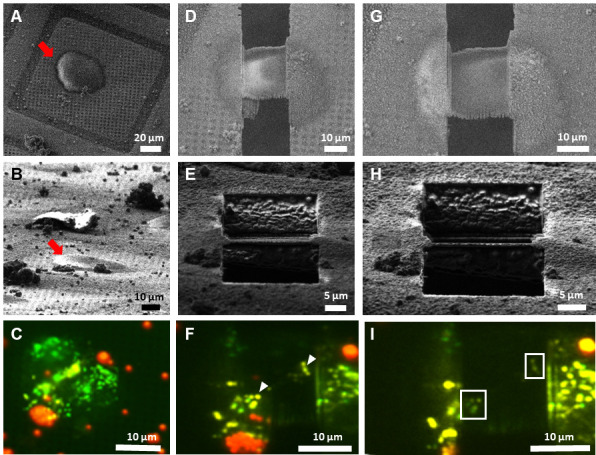
Targeted lamella milling on human RPE-1 cells using METEOR. A–C. Pre-milling status of the cell of interest imaged using SEM, FIB, and METEOR, respectively. Red arrows in SEM and FIB images point toward the same cell. D–F. SEM, FIB, and METEOR images corresponding to a checkpoint at 500 nm lamella thickness, respectively. The white arrowheads point towards fluorescence emitted from potential ROIs. G–I. SEM, FIB, and METEOR images corresponding to the final checkpoint, respectively. White boxes in (I) outline the fluorescent areas in the final lamella.

**Figure 8.**
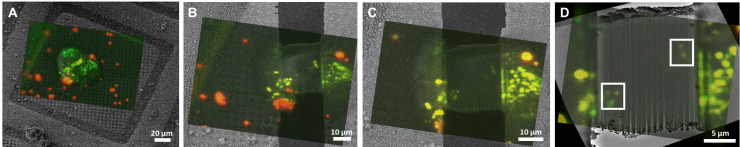
In-situ correlative cryo FIB milling on human RPE-1 cells. Superimposed images after correlation between (A) SEM and METEOR images prior to milling, (B) at 500 nm lamella thickness, and (C) at final lamella thickness. (D) The final fluorescence image of the lamella was additionally correlated with a TEM overview image to pinpoint the suitable locations for tomogram acquisition (white boxes).

**Figure 9.**
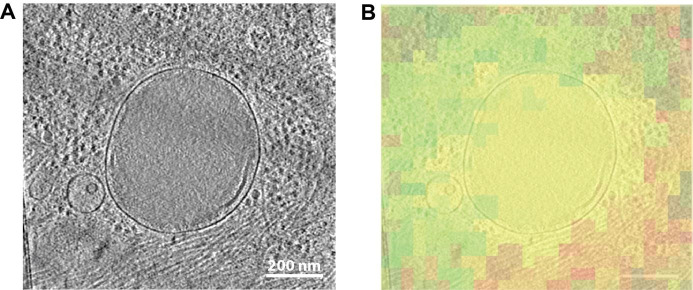
Tomographic slice correlated to METEOR image. A. Image slice from a tomogram showing a lysosome. B. The corresponding fluorescent microscopy image from the METEOR system overlaid transparently with the tomographic slice (red: mCherry-p62; green: autofluorescent background signal, which often accompanies lysosomes).

Figure 7 depicts the step-by-step targeted lamella milling process described in Section 3 of the procedure. Once the cell of interest was chosen based on the in-situ fluorescence signal provided by METEOR, as presented in Figure 7A–7C, the milling process was initiated. To avoid milling away the suitable ROIs, multiple checks were performed during the milling process. In Figure 7D–7F the checkpoint at 500 nm lamella thickness is shown as it confirmed the presence of potential ROIs, allowing for further thinning of the lamella. Once the lamella reached the desired thickness, a final check was performed by METEOR, as indicated in Figure 7G–7I, to ascertain that the lamella is a suitable candidate for tilt series acquisition. Correlations between SEM and FM images at different stages of cryo FIB milling are superimposed to monitor progress as displayed in Figure 8A–8C. This final fluorescence image was additionally correlated with a TEM overview image of the final lamella to accurately pinpoint the ROIs as shown in Figure 8D. The image correlation was used to record tilt series on the sites of interest in the cryo-TEM. Subsequently, the tilt series were reconstructed to yield tomograms and the presence of the ROI was confirmed by correlating the METEOR image to the final tomogram (Figure 9).

## General notes and troubleshooting

**General notes**

By combining PRIMO (Alvéole) with the CERES ice shield (Delmic) and the METEOR (Delmic), the productivity of our cryo-ET workflow improved by an order of magnitude compared to the traditional workflow (Table 1). First, by enhancing the grid quality using the PRIMO photopatterning system, we were able to improve the cell positioning by confining the cells to the center of the grid squares, thereby increasing the number of amenable cells for milling by at least 4-fold. Micropatterning also provides control over the cell shape and hence allows for repositioning of cellular features of interest, e.g., when they are located in the periphery of the cell. Subsequent addition of the CERES ice shield drastically reduced the rate of surface ice contamination build-up in the cryo FIB–SEM chamber. This contamination trap allowed for longer milling sessions, resulting in increased throughput. Finally, by including the METEOR in-chamber fluorescence microscope, we reduced the number of sample transfer steps between instruments, which are the main source of ice contamination. Additionally, the METEOR system allowed for frequent checking of the lamella to make sure that it still contained the ROI. At each intermediate checkpoint, when the ROI was no longer present in the lamella and was milled away, the polishing step on this lamella could be skipped and valuable microscope time could be saved for more suitable sites. Together, the three described integrated additions to the standard FIB–SEM instrumentation are of highly complementary value and improve the quality as well as the efficiency of lamella generation for in situ structural biology approaches.

**Troubleshooting**

Problem 1: No patterns observed and cells growing everywhere on the grid.

Solution 1: Glow discharging may have been inefficient. Increase the glow discharging duration or current. Make sure that you see liquid spreading out on the surface of the grids and not rounding up and rolling off the sides.

Solution 2: The anti-fouling layer/passivation was not carried out under the right conditions as described above. Increase PLL-g-PEG concentration or incubation duration.

Solution 3: Ensure the grid does not dry out while handling it. Drying the grids will cause the anti-fouling layer to collapse.

Solution 4. Reduce incubation time of the cells on the grid. When overgrowing cells on the grid, the cells will continue dividing and eventually spread to non-patterned areas.

Solution 5. Cells could be growing under the grid and not on top of the grid; move the grid to a clean dish with media and check cell distribution on the grid. Always ensure the grids are flat on the bottom of the dish before seeding the cells on top.

Solution 6: After micropatterning, remove the grids immediately from the PLPP photoinitiator solution and wash thoroughly. Extensive incubation after patterning can lead to formation of free radicals that can degrade the PLL-g-PEG layer.

Problem 2: No patterns observed and cells not growing anywhere on the grid.

Solution 1: Increase fibronectin concentration or incubation duration before seeding the cells.

Solution 2: Switch to another extracellular matrix protein such as vitronectin and see if cell growth is not observed.

Solution 3: Ensure the grid material is not toxic to your cells. For instance, copper grids leak ions in the media leading to cell death.

Problem 3: Extensive ice contamination is seen on the lamella despite the CERES ice shield.

Solution 1: Take extreme care while unloading the grid from the cryo FIB–SEM and loading the grid in the TEM. Use only clean liquid nitrogen, work in a low humidity room, work fast but accurately, and, finally, consider wearing a face mask while handling the grid.

Solution 2: Check the cryo FIB–SEM chamber vacuum. Normal wear and tear can lead to leaky tubing and degraded chamber vacuum values.

Problem 4: Extensive devitrification is seen on the lamellae imaged with the METEOR system.

Solution 1: Reduce the excitation light power and/or exposure time.

Solution 2: Always ensure all tools are fully cooled to liquid nitrogen temperatures before use, especially tweezers that are used to handle the grid directly.

Problem 5: No fluorescence is seen in the lamellae using the METEOR system despite the target being present in the lamella.

Solution 1: Increase the excitation power and/or exposure time and use camera binning.

Solution 2: Proteins with low abundance or faint tags may be hard to detect in the final ~150 nm thick lamella. Switch to a newer generation of brighter fluorophores, encode a triple tag, i.e., 3× eGFP instead of eGFP, or consider tagging another more abundant protein that will also localize to your ROI.
